# Elucidating the function of clusterin in the progression of diabetic kidney disease

**DOI:** 10.3389/fphar.2025.1573654

**Published:** 2025-05-14

**Authors:** Lin Mao, Ruili Yin, Longyan Yang, Dong Zhao

**Affiliations:** Beijing Key Laboratory of Diabetes Prevention and Research, Center for Endocrine Metabolic and Immune Diseases, Beijing Luhe Hospital, Capital Medical University, Beijing, China

**Keywords:** diabetic kidney disease, clusterin, glucose metabolism, lipid metabolism, oxidative stress

## Abstract

Diabetic kidney disease (DKD) is a common microvascular complication and the main cause of death in diabetic patients. Metabolic disorders can accelerate the occurrence and development of DKD through a variety of ways, Recent studies have found that Clusterin (Clu) levels are associated with renal dysfunction and can be used as a biomarker of renal tubular injury, while preclinical studies reveal its renoprotective function. This article reviews the molecular mechanisms of Clu in the interaction between various cells in DKD. In addition, we discuss the latest research progress of Clu in the field of DKD. This review aims to explore Clu as a potential therapeutic target for DKD and provide some guidance for future clinical treatment.

## 1 Introduction

Diabetic kidney disease (DKD) is one of the main causes of end-stage renal disease (ESRD) worldwide, which represents the final phase of severe renal failure, with approximately 40%–50% of ESRD cases progressing from DKD (Johansen et al., 2021; Ruiz-Ortega et al., 2020). According to the report released by International Diabetes Federation (IDF) in 2023, the number of new cases of chronic kidney disease (CKD) caused by type 2 diabetes (T2DM) in the world increased by 1 million from 1990 to 2017, an increase of about 74%. Among ESRD patients, the prevalence of diabetes increased by 10.7% from 2000 to 2015 (Cheng et al., 2021). The economic burden of DKD on global healthcare systems is substantial (Ogurtsova et al., 2017; Parker et al., 2024). Previous research has established that individuals with DKD face a significantly heightened risk and mortality rate related to cardiovascular disease. Interestingly, while there has been a decline in the incidence of cardiovascular diseases over the past three decades, this improvement has not been observed in DKD patients (Nathan et al., 2005; Filippatos et al., 2021; Scilletta et al., 2023). The primary pathological changes observed in DKD include glomerulosclerosis, tubular atrophy, and interstitial fibrosis, which are causes of renal failure (Webster et al., 2017).

Currently, clinical treatment options for DKD are quite limited. Conventional approaches primarily focus on delaying disease progression through blood pressure management and glycemic management; however, these methods cannot prevent the onset of DKD (Ruggenenti et al., 2010; Hu et al., 2023). Notably, over 90% DKD patients remain asymptomatic for extended periods before advancing to irreversible renal fibrosis, which often leads to inadequate kidney management among many individuals (Alfego et al., 2021). This oversight contributed to around five million deaths attributed to DKD in 2015 (Tang and Yiu, 2020). Research indicates that 20%–40% of DKD patients develop end-stage clinical symptoms, leaving them with limited treatment options, often restricted to dialysis or renal replacement therapy (Cefalu et al., 2016). The 2023 IDF report shows that only 27%–53% of ESRD patients worldwide are able to receive renal replacement therapy, while low- and middle-income countries are unable to receive renal replacement therapy. Given the unclear specific molecular mechanisms underlying DKD, there is an urgent need to identify new targets for its prevention and treatment.

Clusterin (Clu) is a highly conserved heterodimeric glycoprotein that is present in nearly all tissues and body fluids of mammals, particularly abundant in liver (Seo et al., 2020), brain (Liu et al., 2022a), testis (Xiao et al., 2024), and epididymis (Saewu et al., 2017). Clu exists in multiple isoforms with distinct functions, primarily categorized into two forms: non-glycosylated nuclear variants (nClu) and glycosylated secretory variants (sClu) (Jones, 2002). The secretory variant, sClu, functions as a molecular chaperone and has been shown to confer protective effects in various models of acute and chronic kidney injury (Pais et al., 2023; Jung et al., 2012). Current studies suggest that nClu is predominantly localized in the nucleus following exposure to ionizing radiation, although its abundance remains relatively low (Shannan et al., 2005). Thus, there is ongoing debate regarding the role of Clu in pathological processes.

This article aims to review the existing literature on Clu in the context of kidney function, with the objective of laying a theoretical foundation for its potential use as a predictive, therapeutic, and prognostic biomarker in DKD.

## 2 The function of Clu in DKD

### 2.1 The progression of DKD

According to the latest DKD report from the IDF, the incidence of CKD associated with T2DM in worldwide increased by 74% between 1990 and 2017 (Federation, 2023). It is projected that by 2030, the number of diabetes patients transitioning to ESRD will steadily reach approximately 4.35 million, representing an increase of 1.531 million compared to 2010 (Liyanage et al., 2015).

Currently, the key clinical indicators for assessing renal function include an estimated glomerular filtration rate (eGFR) of less than 60 mL/min/1.73 m^2^ or proteinuria exceeding 30 mg/g, along with sustained proteinuria greater than 300 mg over 24 h (Rossing et al., 2022). However, the lack of more non-invasive biomarkers to evaluate renal function imposes limitations on the clinical assessment of early renal injury and disease progression. Therefore, a comprehensive understanding of the pathogenesis of DKD is crucial for delaying, preventing, or reversing disease progression and ultimately improving patient outcomes.

DKD is characterized by several distinctive pathological features, including hypertrophy of glomerular capillaries, thickening of basement membrane, loss of podocyte foot processes, and dilation of mesangial area (Ricciardi and Gnudi, 2021). Notably, early injuries in DKD often manifest before the onset of microalbuminuria, primarily as endothelial dysfunction (Stehouwer, 2004; Lassén and Daehn, 2020) and an elevated eGFR (Ricciardi and Gnudi, 2021).

In the initial stages, there is an increase in both the glucose concentration within the glomerular filtration and the amount reabsorbed by renal tubules. This leads to a decrease in sodium concentration reaching the distal nephron, subsequently promoting relaxation of glomerular arteries (Tonneijck et al., 2017; Stefansson et al., 2022; Cortinovis et al, 2022). This physiological shift stimulates renin secretion from juxtaglomerular cells through a feedback mechanism, elevating angiotensin II level, which in turn induces vasoconstriction of glomerular arterioles. The resulting increase in intraglomerular pressure fosters a state of hyperfiltration (Patinha et al., 2013; Hall et al., 2021).

**FIGURE 1:**
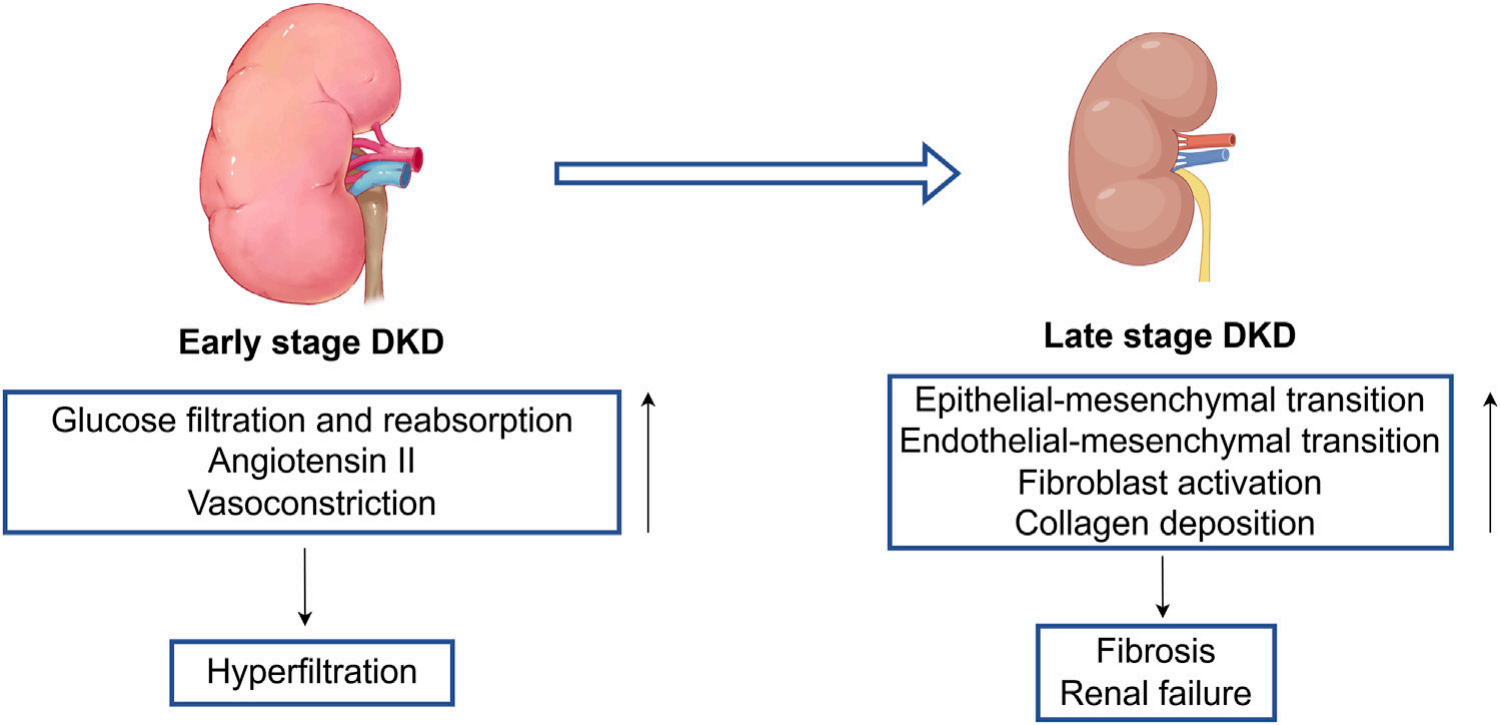
The pathogenesis of early stage to late stage of DKD. Early stage DKD is mainly characterized by increased glucose filtration and reabsorption, expression of angiotensin II, and vasoconstriction, leading to a state of renal hyperfiltration. There are multiple stimulating factors in the late stage DKD that cause endothelial mesenchymal transition and epithelial mesenchymal transition, leading to loss of normal cell function, activation of fibroblasts, accumulation of collagen fibers, and ultimately causing end-stage changes (renal fibrosis and renal failure).

As DKD progresses into later stages, it can lead to significant pathological transformations, including epithelial-to-mesenchymal transition (EMT) (Yang and Liu, 2001; Hertig et al., 2008) and endothelial-to-mesenchymal transition (Li et al., 2009; Jin et al., 2023). This progression is associated with the activation of fibroblasts and collagen deposition within the extracellular matrix (Gibb et al., 2020; Yamashita and Kramann, 2024), disrupting the delicate balance between fibrosis that supports wound healing and fibrosis that inflicts long-term damage to renal structure and function (Liu, 2011). Ultimately, these pathological changes culminate in fibrosis and scar formation (Humphreys, 2018; Moretti et al., 2022), leading to renal failure (Liu, 2011) (Figure 1).

Recent studies have identified Clu as a valuable predictor of renal tubular injury in cases of acute kidney injury induced by drug toxicity (Pais et al., 2023; Pye et al., 2024; Ravindra et al., 2024; Wruck et al., 2022). It is worth noting that in the continuous cohort of T2DM patients with diabetes duration for more than 6 years, urinary Clu concentration increased significantly compared with normal people, which suggests that urinary Clu can be used as a diagnostic marker for DKD and microalbuminuria in diabetic patients (Zeng,et al., 2017). Another study, which included T2DM patients and non-diabetes patients with eGFR ≥ 60 mL/min/1.73 m^2^, showed that urinary Clu was associated with the annual decline rate of eGFR, the development of CKD stage 3 or greater, and the persistence/progress for proteinuria in DKD individuals (Kim et al., 2017). In addition, urinary Clu level can also be used as a new urinary protein biomarker for development of microalbuminuria and decline of renal function in patients with type 1 diabetes (Schlatzer et al., 2012).

The role of Clu in a variety of metabolic diseases (such as non-alcoholic fatty liver disease (Seo et al., 2025; Park et al., 2018; Park et al., 2020), diabetes (Seo et al., 2020; Seo et al., 2018), obesity (Hoofnagle et al., 2010), etc.) has been supported by many research results. DKD is the main cause of renal failure in diabetes patients. Current research has shown a correlation between Clu and various acute and chronic kidney injuries, but in the past 5 years, there has been a lack of in-depth research on the relationship between Clu and DKD. Therefore, it is necessary to fill this gap and clarify the potential significance of Clu in the context of DKD management and assessment.

### 2.2 Description of Clu

Clu is a heterodimeric glycoprotein composed of 449 amino acids, and it is classified as a lectin due to its ability to aggregate blood cells (O Blaschuk, 1983). Clu exhibits considerable homology across different species and is widely expressed in various mammalian tissues and body fluids, with the highest expression levels observed in liver, brain, and testis (Shannan et al., 2005; Wong et al., 2005). The Clu gene is located on chromosome 8p21.1 and consists of nine coding exons and two untranslated exons (Herring et al., 2019). Alternative splicing and post-translational modifications of these coding exons result in the production of multiple Clu isoforms (Wong et al., 2005). There are three principal transcripts of the human Clu gene, distinguished primarily by their different translation initiation sites, which may account for the varied roles Clu plays in different pathological contexts (Bonacini et al., 2015). *In vitro* studies utilizing antisense oligonucleotides (ASOs) that inhibit exon 2 have demonstrated that this exon is closely linked to the N-terminal endoplasmic reticulum localization signal. Under cellular stress conditions, Clu can undergo alternative splicing that skips exon 2, resulting in Clu isoform that is only subjected to core glycosylation (Nizard et al., 2007). Due to the absence of endoplasmic reticulum (ER) leader sequence. Clu accumulates in the cytoplasm (Foster et al., 2019) and affects apoptosis through the BAX pathway (Leskov et al., 2011; Rodríguez-Rivera et al., 2021; Velasco et al., 2013).

Typically, Clu is secreted via the classical ER-Golgi pathway (Li et al., 2012). Initially, a low-glycosylated protein precursor weighing approximately 60 kDa is synthesized, directed to ER by a signaling sequence that facilitates preliminary glycosylation. At this stage, it is referred to as the secretory Clu precursor (Foster et al., 2019). This form of Clu precursor has been found to have a regulatory effect on NFκB activity (Essabbani et al., 2010). Following this, the precursor undergoes further glycosylation in the Golgi apparatus, resulting in a protein with a molecular weight of 70–80 kDa. Proteolytic cleavage by a furin-like enzyme then generates 35–40 kDa α and β subunits (Kapron et al., 2008), which are linked by disulfide bonds to yield the fully mature and secretory form of the protein (Choi-Miur et al., 1992; Rohne et al., 2016; Sabatte et al., 2011).

Secretory Clu functions as a molecular partner that protects cells by clearing excess debris and misfolded proteins (Bell et al., 2007; Wyatt et al., 2011; Wilson and Easterbrook-Smith, 2000). Additionally, Clu enhances cell survival and proliferation, potentially representing another mechanism underlying its cytoprotective effects (Mitsufuji et al., 2022; Nguan et al., 2014). The promoter region of the Clu gene contains regulatory elements associated with transforming growth factor β (TGFβ) (Liu et al., 2022b), activator protein-1 (AP-1), and activator protein-2 (AP-2), as well as elements responsive to oxidative stress (Park et al., 2013). As a chaperone protein, Clu inhibits protein aggregation and precipitation (the main characteristic of oxidative damage), and reduces hydrogen peroxide induced oxidative stress through the PI3K/Akt pathway, helping cells to store beneficial substances. Research indicates that decreased expression of Clu in response to various stressors correlates with markers of cellular aging and redox imbalance, suggesting that Clu may play a role in the oxidative stress response (Chammas et al., 2011). Clu prevents oxidative stress-related liver toxicity through the Akt-Keap1-Nrf2 signaling pathway(Ma et al., 2022).

## 3 Functions of Clu in kidney physiology and pathology

### 3.1 Physiology

Clu is a multifunctional glycoprotein whose expression is regulated in mouse kidneys. There are stage differences in the expression of Clu in the kidneys. Clu is widely expressed in the tubules of newborn mice, while this phenomenon was not observed in the glomeruli. In the late stage of development, only Clu expression was observed in the newborn tubules (mainly in the medulla) of adult mice, which may suggest that Clu plays a role in organ development (Harding et al., 1991; French et al., 1993).

### 3.2 Pathology

Hypoxia is an important factor in renal ischemia-reperfusion (IRI) injury, during which Clu plays a role in promoting renal tissue repair (Zhou et al., 2010). Through transcriptome analysis of the role of Clu in renal tubular epithelial cells under hypoxic conditions, the results showed that Clu mainly promotes cell growth and survival through a cascade mediated by PI3K/Akt, but inhibits cell migration under normoxic conditions (Dairi et al., 2016). Hypoxia causes an increase in Clu secretion in renal mesenchymal stromal cells, then treatment with this conditioned medium promotes cell proliferation and is necessary for regulating M2 polarization and phagocytic activity of macrophages (Weng et al., 2022). In addition, Clu protein expression in monocytes/macrophages also increased. After LPS and IRI, macrophages infiltration in kidney of Clu-KO mice was significantly higher than that WT mice, leading to sustained kidney inflammation and tissue fibrosis (Weng et al., 2020; Guo et al., 2016). Autophagy that promotes cell survival is Clu dependent, and autophagy dysfunction occurs after Clu deficiency (Alnasser et al., 2016). Clu-KO mice showed autoimmune symptoms in kidney, such as antibody production, immunoglobulin and complement component deposition, reduced macrophage clearance of apoptotic cells, and caused autoimmune response induced by apoptotic cells (Cunin et al., 2016).

## 4 Role of Clu in DKD

Non-communicable diseases have become a major threat to global health, accounting for over 70% of global deaths. Cardiovascular-renal-metabolic (CKM) syndrome emphasizes the close association between metabolic function and kidney disease. CKD has the highest prevalence in CKM, affecting 674 million people. Diabetes is the main cause of CKD. 11% of the global population has diabetes, which is expected to reach 783 million in 2045. And about 1/3 of diabetes patients have CKD as a complication. Therefore, elucidating the role and mechanism of Clu on DKD can help us gain a deeper understanding of DKD and identify targets for its prevention and treatment (Xie et al., 2025). Bioinformatics analysis of differentially expressed genes between normal kidney tissue and DKD kidney tissue showed that Clu is involved in the occurrence and development of DKD (Xu et al., 2021). Clu expression has been detected in both glomeruli and tubular cells. A recent study examining the interaction of Clu between podocytes and renal tubules found that diabetes enhances the expression of transcription factor KLF6 in podocytes, subsequently increasing the secreted Clu, which in turn activates CaMK1D signaling by specifically binding to the low-density lipoprotein receptor associated protein 2 (LRP2) on proximal tubular epithelial cell (PTEC) membrane, thereby improving mitochondrial function (Gujarati et al., 2024).

### 4.1 Effect of sClu on DKD

Clu gene polymorphism was significantly associated with the prevalence of T2DM (Daimon et al., 2011). In a 2021 whole-genome study of patients with T2DM and DKD, it was found that Clu is an oxidative stress gene related to DKD, particularly the variant at rs11780592 (p = 0.013), is a risk factor for DKD progression. Conversely, the variant at rs7824575 (p = 0.039) appears to confer a protective effect against DKD (Roumeliotis et al., 2021). There is no difference in circulating sClu levels between normal populations of different genders (Kujiraoka et al., 2006). Studies have shown that circulating sClu levels are significantly elevated in obese individuals compared to their lean counterparts, and these levels correlate positively with body mass index (BMI), waist circumference, inflammatory markers, and indicators of insulin resistance (Werida et al., 2021; Wang et al., 2014; Moro et al., 2014). Further research was conducted on the Clu content in high density lipoprotein (HDL) of subjects with lean insulin sensitivity, lean insulin resistance, and obese insulin resistance. Compared with the lean insulin sensitivity group, the HDL-Clu content in the lean insulin resistance group was significantly reduced, while the content of obese insulin resistance group was lower (Hoofnagle et al., 2010). This suggests that circulating sClu is positively correlated with insulin resistance, metabolic syndrome and diabetic courses, while HDL-Clu is negatively correlated with the above indicators. In addition, serum sClu levels in pre-diabetes and diabetes patients were increased, and were independently related to adipose tissue insulin resistance (Adipo-IR) (Croyal et al., 2022; Wang et al., 2023).

Prior research indicates that Clu enhances the sensitivity of leptin receptor-mediated signaling through interactions with LRP2, emphasizing its role in metabolic regulation (Bartl et al., 2001; Gil et al., 2013). Furthermore, a reduction in circulating Clu concentrations has been observed following 2 weeks on a calorie-restricted diet. Clu reduction on low-density lipoprotein (LDL) particles is associated with LDL aggregation, which may be the reason for affecting circulating lipid levels (Ruuth et al., 2021). The full-length Clu protein consists of two chains, α and β, which have differential effects on lipid metabolism. Specifically, full-length Clu reduces lipid levels while the β chain is associated with an approximate 40% increase in body weight, obesity-related adipocyte hypertrophy, and hepatic and renal steatosis-effects not noted with the α chain alone (Matukumalli et al., 2017).

Beyond its role in lipid metabolism, Clu also participates in glucose homeostasis, as indicated by the presence of two E-box motifs in intron 1 that function as high-glucose response elements regulated by SREBP-1c (Kim et al., 2011). Clu-LRP2 axis of liver-muscle is crucial for maintaining normal glucose homeostasis and insulin sensitivity (Seo et al., 2020). Genetic polymorphism studies have identified that the intron 7 Clu variant at rs2279590 is associated with insulin resistance (HOMA-IR) and disorders of insulin secretion (HOMA-β) among diabetes patients (Valko et al., 2007). Elevated expression of Clu has been noted in skeletal muscle and liver of mice fed a high-fat diet, while systemic knockout Clu resulted in increased insulin sensitivity compared to wild-type mice (Park et al., 2014).

**FIGURE 2:**
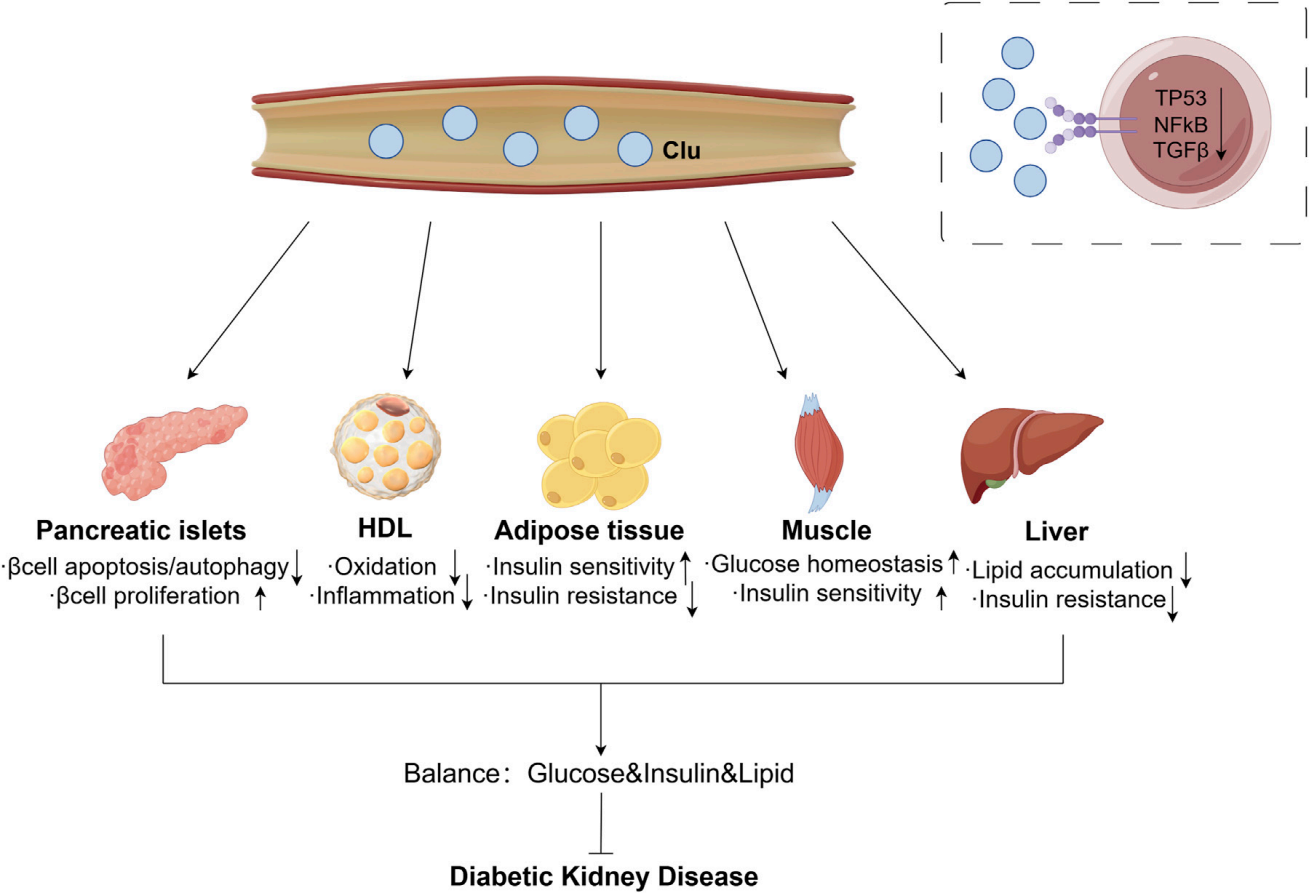
Effect of circulating sClu on DKD. Circulating sClu may alleviate DKD through the following ways: (1) Pancreatic islets: reduce β cell apoptosis and autophagy, promote βcell proliferation; (2) HDL: reduce inflammation by inhibiting HDL oxidation; (3) Adipose tissue: increase insulin sensitivity and reduce insulin resistance; (4) Muscle: increase muscle homeostasis to glucose and insulin sensitivity; (5) Liver: reduce lipid accumulation and insulin resistance. Clu, clusterin; HDL, high density lipoprotein; TP53, tumor Protein 53; NFκB, nuclear factor kappa B; TGFβ, transforming growth factor β.

Moreover, circulating sClu may improve the damage caused by metabolic abnormalities to protect pancreatic beta cells. The serum of different groups of participants (young and old, non-obese and obese, non-diabetes and diabetes) after 4 and 8 weeks of exercise or direct Clu treatment have protective effects on β cells apoptosis induced by severe ER stress, which can last up to 2 months after the end of training program (Coomans de Brachène et al., 2022). Another study also demonstrated that Clu protects pancreatic beta cells from free fatty acids (FFA)-induced lipotoxicity apoptosis by promoting LC3-II mediated autophagy (Hong et al., 2020). In diabetic environment, islet α cells overexpress Clu, then promote the proliferation of β cells through paracrine form (Kim et al., 2001). Summary of circulating sClu impacts DKD is shown in Figure 2.

The above studies consistently suggested that upregulation of Clu expression in disease states may be a self-protection response, suggesting that this theory also holds true in DKD. The upregulation of Clu in DKD is to alleviate kidney damage, but its own secretion is not sufficient to completely prevent disease progression. Therefore, regulating the expression or function of Clu may help enhance this protective effect, thereby delaying the progression of DKD. However, the effects of sClu released from extrarenal tissues on kidney function remain poorly understood, highlighting the need for further research to elucidate its role and underlying mechanisms in renal pathology.

### 4.2 Effect of Clu in renal cells on DKD

#### 4.2.1 Endothelial cell

The severity of DKD in T2DM patients has been positively correlated with endothelial dysfunction (Sun et al., 2023). Glomerular endothelial cells serve as a crucial component of the glomerular filtration barrier, acting as the first line of defense between blood vessels and blood flow. When these endothelial cells come into direct contact with harmful circulating substances, injury ensues, leading to increased protein excretion in urine. In patients with diabetes, the amount of Clu bound to HDL in endothelial cells increases, however, due to oxidative modifications and glycosylation, the cytoprotective effects of Clu are compromised (Sanda et al., 2021). The antioxidant compound aminoguanidine (AG) has been shown to reduce Clu expression levels induced by IRI, while concurrently enhancing the mRNA expression of renal medullary endothelial markers such as VE-cadherin and CD31 (Kovacic et al., 2012; Pasten et al., 2021). Lycopene supplementation can prevent LDL induced coronary endothelial dysfunction by mitigating oxidative damage, enhancing endothelial nitric oxide synthase (eNOS) expression and activity, and improving the antioxidant capacity of HDL (Vilahur et al., 2015). In a hyperglycemic environment, plasma analysis from diabetic patients revealed a significant reduction in Clu protein levels, accompanied by an increase in endothelial cell apoptosis rates by 1.4–2.3 times (Liu et al., 2014). Elevated levels of plasma FFA have been found to decrease Clu expression—both at mRNA and protein levels—by up to 54% in a concentration-dependent manner. Additionally, endothelial cell apoptosis rates have increased by 4.2-fold in samples from patients with high concentrations of FFAs (Artwohl et al., 2003). Under diabetic conditions, increased shear stress can upregulate Clu expression, thereby counteracting complement-induced inflammation in endothelial cells and preventing their activation (Urbich et al., 2000). Moreover, the application of recombinant Clu protein to endothelial cells has shown protective effects against oxidative injury and has been demonstrated to restore tight junction integrity in endothelial cells under diabetic conditions (Kim et al., 2009; Kim J. H. et al., 2010).

#### 4.2.2 Mesangial cell

During both acute and chronic kidney injury, fibrin—a product of thrombin activity—accumulates in kidney, leading to a 2 to 4-fold increase in the expression of Clu mRNA in glomerular mesangial cells, glomerular epithelial cells, and PTECs (Laping et al., 1997). *In vitro* and *in vivo* studies have demonstrated that Clu expression is upregulated in rat glomerular mesangial cells following complement-mediated injury, particularly associated with deposition of the complement activation complex C5b-9 (Yamada et al., 2001). Clu is known to be induced in various injury contexts and accumulates at sites of tissue remodeling and degeneration. Notably, studies involving knockout Clu mice have revealed significant insights into its protective role. In aging mice lacking Clu, deposition of immune complexes was observed in the glomerular mesangial cells, and by 21 months of age, approximately 75% of the glomeruli exhibited moderate to severe mesangial lesions. This suggests that Clu plays a crucial protective role in maintaining glomerular integrity (Rosenberg et al., 2023). Additionally, as male rats age, Clu mRNA expression in the renal cortex increases, accompanying a higher apoptosis rate in mesangial cells of older male rats compared to their younger counterparts (P C Singhal, 1997). Following high-fat feeding, knockout Clu mice exhibited pronounced mesangial dilation, fibrosis, and increased urinary albumin-creatinine ratio (UACR). In Zucker obese rats, there is significant glomerular mesangial dilation, loss of podocyte foot processes, and interstitial fibrosis, with urinary levels of Clu markedly elevated compared to controls. Treatment with valsartan not only ameliorates the pathological alterations in glomeruli but also reduces urinary Clu levels. In diabetic mice, an increase in sClu levels was observed in both glomeruli and renal tubules. The level of sClu in DKD mice increased, and nClu in podocytes, mesangial cells and damaged tubular cells related to apoptosis also increased (Tunçdemir and Ozturk, 2008). These findings underscore the complex role of Clu in kidney pathology, particularly in the context of diabetes and obesity-related kidney injury.

#### 4.2.3 Podocyte

Podocytes are specialized, non-renewable epithelial cells that play a crucial role in the integrity of the glomerular filtration barrier (Jefferson et al., 2008). In T2DM patients, a reduction in podocyte number and detachment of podocytes from the glomerular basement membrane are associated with albuminuria (Chen et al., 2008; Yaoita et al., 2014). Both glucose toxicity (Katalin Susztak et al., 2006) and lipid toxicity (Kim et al., 2021) in DKD adversely affect the mitochondrial and ER functions of podocytes, resulting in increased reactive oxygen species (ROS) production, oxidative stress, inflammatory response, and insulin resistance. After acute kidney injury (AKI), urinary Clu levels rise significantly, particularly 72 h post-surgery. Collecting urine from patients and culturing podocytes have been shown to activate TP53 and SIRT1, helping to maintain a balance among proliferation, angiogenesis, and cell cycle arrest, thus facilitating the repair of damaged nephrons (Erichsen et al., 2023). The anti-aging compound CMS121 has been found to decrease urinary Clu levels in mice while also improving urinary markers of kidney injury (Zahid et al., 2023). In models simulating renal injury, such as chimeric bovine serum albumin (cBSA), knockout Clu mice displayed elevated serum creatinine and proteinuria, alongside severe glomerular atrophy, mesangial dilation, and C3 deposition in kidney (Rosenberg et al., 2023). Over time, glomerular lesions in these Clu-deficient mice progressively worsened.

While sClu is recognized as a complement inhibitor, it does not exert inhibitory effects on complement activation at physiological concentrations (Hochgrebe et al., 1999). However, in pathological conditions, sClu forms a complex with complement component SC5b-9 and membrane attack complex (MAC), reducing kidney inflammation and damage by preventing complement-dependent podocyte degeneration (Sun et al., 2020) and preventing MAC-mediated podocyte injury (Murphy et al., 1988; Tschopp and French, 1994). In autoimmune glomerular diseases, Clu co-localizes with LDL receptors. Moreover, pre-incubation of podocytes with recombinant Clu has been shown to inhibit the upregulation of phosphorylated PKC (pPKC) α/β and reduce cellular inflammation. Interestingly, multivariate analysis indicates that glomerular Clu expression is a significant factor influencing proteinuria, with a correlation observed after 1.5 years of follow-up (p = 0.027) (Rastaldi et al., 2006).

In both DKD patients and DKD murine models, an increase in Clu expression within the glomeruli has been noted. Additionally, pre-treatment of podocytes with recombinant human Clu significantly reduces oxidative stress-induced apoptosis, ultimately improving cell viability (He et al., 2020). Furthermore, two different diabetic rat models—Zucker obese rats and ZDSD rats—exhibited podocyte loss, increased proteinuria, and elevated urinary Clu levels (Habibi et al., 2019; Peterson et al., 2017). Given the distinctive roles of podocytes, further research is warranted to elucidate the specific mechanisms by which Clu functions in these cells.

#### 4.2.4 Tubular epithelial cell

In DKD models, damage to PTECs has been observed to occur prior to declines in renal function (Li et al., 2021). These damaged tubular cells release a variety of inflammatory and fibrotic mediators, which drive interstitial inflammation and fibrosis—key pathological processes that contribute to the progression of DKD to ESRD (Zhang et al., 2023; Lu et al., 2019). This suggests that renal tubular injury may precede glomerular damage, acting as a critical driver in the onset and progression of DKD (Li et al., 2022; Xie et al., 2022). Studies have identified that Clu was a key gene in tissue samples of normal people and DKD patients with renal tubular injury, and its expression increased (Yang et al., 2022).

In the rat models of autosomal dominant polycystic kidney disease and focal segmental glomerulosclerosis (FSGS) after bilateral renal ischemia, urinary Clu levels were significantly elevated in autosomal dominant polycystic kidney disease rats, whereas FSGS rats did not show increased urinary Clu despite having more severe proteinuria (Hidaka et al., 2002), which may help distinguish between tubular and glomerular forms of proteinuria. In the context of lupus nephritis, Clu accumulation was primarily localized in the renal tubular epithelial cells (RTECs), with minimal presence in the affected glomeruli (Solange Moll et al., 1998). Notably, Clu mRNA expression was found to be 8.5 times higher in the kidneys of rats with glomerular lesions, with immunohistochemical analysis showing its primary presence in the dilated tubules of both the cortex and medulla (Correa-Rotter et al., 1998; Laping et al., 1998).

In patients with diabetes and a disease duration exceeding 6 years, the ratio of Clu to urinary albumin-creatinine showed a positive correlation, and multivariate analysis indicated that urinary Clu levels were associated with CKD stages 3 and above, as well as the progression of proteinuria (Zeng et al., 2017; Kim et al., 2017). SGLT-2 inhibitors have been shown to diminish Clu expression induced by renal inflammation and oxidative stress, ultimately improving kidney function (Ali et al., 2019).

Clu is not only implicated in cell death (Schlegel et al., 1992; Bajaj et al., 2020) but also colocalized spatially with apoptotic proteins (such as apoptosis-inducing factor and cleaved caspase-3) and autophagy-related proteins (like LC3-II and p62) (Sansanwal et al., 2015). Furthermore, exposure to BSA in PTECs activates inflammatory cytokines and reduces the expression of the anti-apoptotic protein Bcl-xL, leading to a marked increase in Clu levels in culture medium, knockout Clu reverses these effects (Takase et al., 2008). Following ischemia-reperfusion injury, Clu expression is upregulated in PTECs and their culture medium, contributing to reduced cell apoptosis. Nephrotoxic agents can elevate Clu levels in RTECs and urine, and Clu has been demonstrated to mitigate cytotoxicity through mechanisms that are dose-dependent and independent of megalin, a known Clu receptor, it does not confer protection in ATP-depleted cells (Girton et al., 2002). In Clu knockout mice, kidney damage was more severe after 30 days compared to control mice, characterized by elevated serum creatinine and blood urea nitrogen (BUN) levels, increased infiltration of inflammatory cells, renal tubular injury, and tissue fibrosis, resulting in poorer recovery from kidney injury (Zhou et al., 2010; Guo et al., 2016). During the renal repair phase of ischemia-reperfusion injury, Clu in RTECs is associated with genes related to cell cycle and DNA damage repair, promoting cell proliferation and providing renal protection (Nguan et al., 2014). Importantly, during hypoxic conditions, Clu activates the unfolded protein response (UPR) and ER stress pathway, promoting cell survival (Dairi et al., 2016). Autophagy, an additional mechanism that supports cell survival, also relies on Clu under hypoxic circumstances, with its protective effects linked to UPR activation (Alnasser et al., 2016).

Overexpression of Clu in RTECs has been shown to diminish the expression of TGFβ-induced fibrosis related proteins by inhibiting Smad3 phosphorylation and its subsequent nuclear translocation. This anti-fibrotic effect of Clu appears to correlate with localized concentrations in kidney rather than being mediated by Clu secreted from liver (Jung et al., 2012). Additionally, renal cystine poisoning is characterized by proximal tubular dysfunction, with low or absent Clu levels, which would otherwise exert a protective effect on cystine-affected cells. In DKD models, both *in vivo* and *in vitro*, upregulation of Apoc1 leads to downregulation of Clu have been observed, suggesting that knockdown Apoc1 can mitigate high glucose-induced oxidative stress and apoptosis in RTECs, thereby providing a protective effect against DKD (Chai et al., 2024).

**FIGURE 3:**
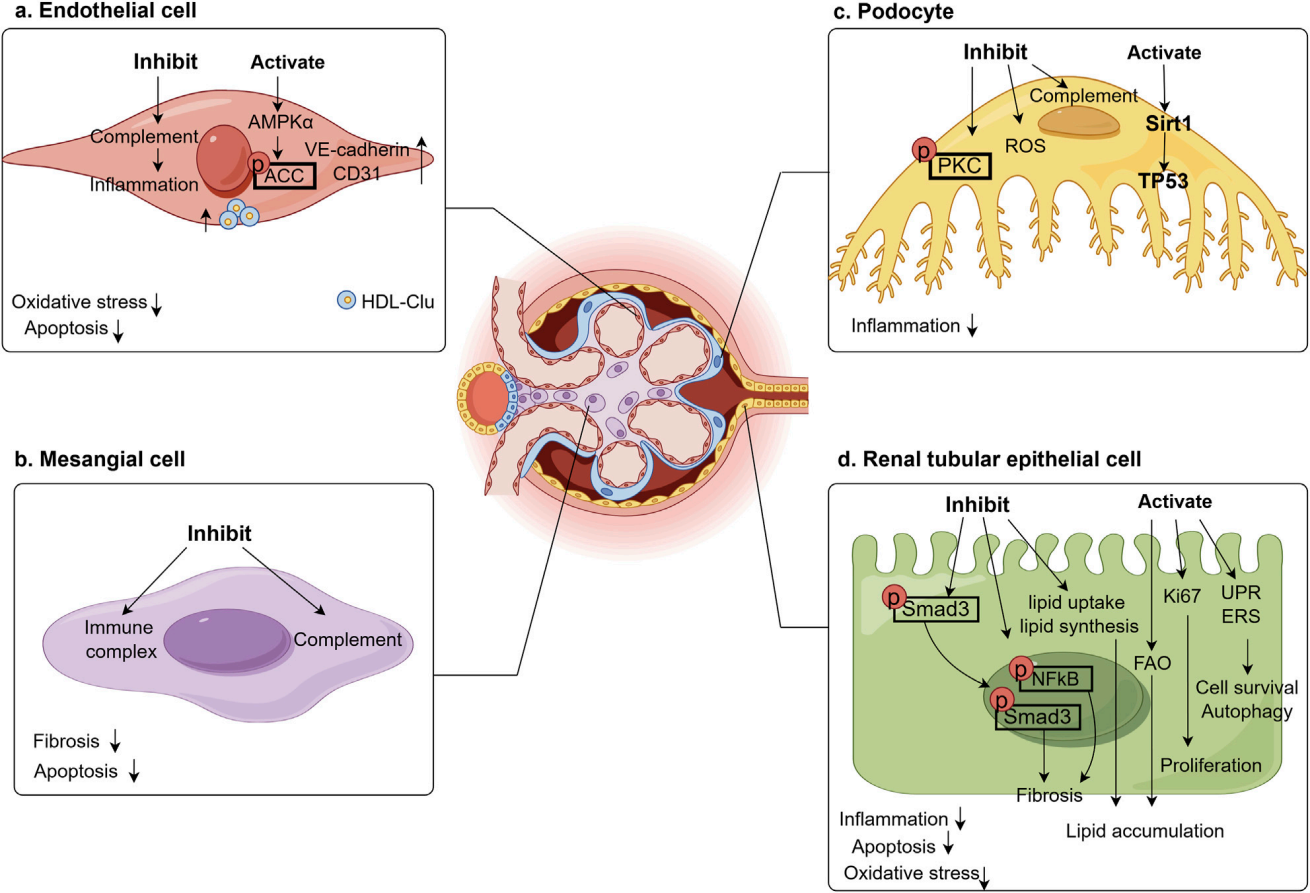
Effect of Clu in renal cells on DKD. Clu plays a renal protective role in various kidney cells, improving oxidative stress, inflammation, apoptosis, and fibrosis in the kidneys. **(a)** Clu inhibits complement activation in glomerular endothelial cell, activates AMPK to promote ACC phosphorylation, and increases endothelial cell markers, ultimately reduces endothelial cell oxidative stress, inflammation, and apoptosis. AMPKα, adenosine 5′-monophosphate (AMP)-activated protein kinase α; ACC, acetyl-CoA Carboxylase; VE-cadherin, vascular epithelial cadherin; CD31/PECAM1, platelet endothelial cell adhesion molecule1; HDL-Clu, high density lipoprotein-clusterin; **(b)** Clu inhibits immune complex and complement in glomerular mesangial cell, reduces cell apoptosis and renal fibrosis. **(c)** Clu inhibits complement and ROS in glomerular podocyte, reduces PKC phosphorylation, activates Sirt1 to increase TP53 expression, finally reduces inflammation. PKC, protein kinase C; ROS, reactive oxygen species; Sirt1, sirtuin 1; TP53, tumor Protein 53; **(d)** Clu reduces the nuclear translocation of phosphorylated Smad and the expression of phosphorylated NFκB in renal tubular epithelial cell, inhibits lipid uptake, synthesis and activation of fatty acid oxidation to reduce lipid accumulation. It also activates ki67, endoplasmic reticulum stress response, and unfolded protein response to promote cell survival and proliferation, reduces inflammation, oxidative stress, and cell apoptosis. Smad3, small mothers against decapentaplegic 3; NFκB, nuclear factorkappa B; FAO, fatty acid oxidation; UPR, unfolded protein response; ERS, endoplasmic reticulum stress.

Moreover, Clu has been shown to prevent oxidative stress in RTECs induced by both exogenous and endogenous factors (Schwochau et al., 1998). High concentrations of TGFβ1 lead to increase lipid uptake and triglyceride synthesis in kidney while decreasing fatty acid oxidation, resulting in lipid accumulation within RTECs. Treatment with adenoviruses or recombinant Clu protein significantly suppressed the expression of adipogenic proteins and lipid levels in these cells (Heo et al., 2018). Furthermore, the oral adsorbent AST-120 was found to reduce renal Clu expression in a unilateral nephrectomy rat model, thereby ameliorating tubulointerstitial injury (Aoyama et al., 2002). In Clu-deficient mice, Angiotensin II (AngII) stimulation exacerbated renal fibrosis and elevated angiotensin type 1 receptor (AT1R) expression. Supplementation with Clu appeared to counteract this by reducing the nuclear localization of phosphorylated NFκB in AngII-induced RTECs, inhibiting NFκB activation, and downregulating AT1R levels, thereby lessening the extent of renal fibrosis (Ashton et al., 2014). Further emphasizing the central role of Clu within renal tubular epithelium. Figure 3 summarizes the role of Clu in different renal cells on DKD.

#### 4.2.5 Fibroblast

Renal fibrosis represents the primary pathological change associated with decreased renal function in patients with ESRD. Clu has been identified in both liver stellate cells (Seo et al., 2019) and lung fibroblasts (Peix et al., 2018; Kim T. H. et al., 2010). Current research results have confirmed that Clu plays a role in renal fibrosis (Jung et al., 2012; Guo et al., 2016; Ashton et al., 2014), but its specific relationship with renal fibroblasts remains unexplored. A crucial mechanism underlying the process of renal fibrosis is the activation of renal interstitial fibroblasts into myofibroblasts in response to various stimulus. Notably, in Clu-deficient mice, there is a significant upregulation of fibrosis-related gene expression (CCL12, Col3a1, MMP9 and TIMP1) in kidney. Additionally, CCL12 has been shown to stimulate the proliferation of fibroblasts and myofibroblasts, which may be due to the phenotypic transformation of fibroblasts and an increase in myofibroblast population caused by knocking out Clu, leading to the aggravation of renal fibrosis (Guo et al., 2016). Recombinant Clu can inhibit enzyme activity by interacting with soluble form of MMP25, a member of membrane matrix metalloproteinase (MMP) family. This suggests that Clu may serve as a negative regulator of MMP family enzyme activity *in vivo*, regulating neutrophil function and preventing excessive tissue damage (Matsuda et al., 2003). Further research is needed in future to clarify the specific mechanism by which Clu exerts anti-fibrotic effects in renal fibroblasts.

The current research suggests that Clu could have a physiologically appropriate but insufficient response in the context of fibrosis, exerting a renal protective effect by limiting the degree of fibrosis. More studies are needed in future to confirm the role and specific mechanism of Clu in renal fibroblasts.

## 5 Conclusion

Clu is emerging as a promising biomarker characterized by its non-invasive and easily accessible nature, offering valuable technological support for the early diagnosis and management of kidney injury in DKD patients, as well as for monitoring disease progression. If future research can establish the dynamic range of Clu variations across different stages of DKD severity, it may become feasible to utilize circulating or urinary Clu concentrations in clinical settings to assess renal function. This advancement could enhance clinical management strategies and improve treatment outcomes for DKD patients. In the future, larger scale cohort studies, clinical trials, and basic research are needed to validate the role and specific mechanisms of Clu in DKD.
